# Prospective patient stratification into robust cancer‐cell intrinsic subtypes from colorectal cancer biopsies

**DOI:** 10.1002/path.5051

**Published:** 2018-03-25

**Authors:** Matthew Alderdice, Susan D Richman, Simon Gollins, James P Stewart, Chris Hurt, Richard Adams, Amy MB McCorry, Aideen C Roddy, Dale Vimalachandran, Claudio Isella, Enzo Medico, Tim Maughan, Darragh G McArt, Mark Lawler, Philip D Dunne

**Affiliations:** ^1^ Centre for Cancer Research and Cell Biology Queens's University Belfast Belfast UK; ^2^ Department of Pathology and Tumour Biology, Leeds Institute of Cancer and Pathology St James Hospital Leeds UK; ^3^ North Wales Cancer Treatment Centre Rhyl UK; ^4^ Centre for Trials Research Cardiff University Cardiff UK; ^5^ Countess of Chester Hospital Chester UK; ^6^ University of Torino, Department of Oncology Candiolo, Torino Italy; ^7^ Candiolo Cancer Institute, FPO‐IRCCS Candiolo, Torino Italy; ^8^ CRUK/MRC Oxford Institute for Radiation Oncology University of Oxford Oxford UK

**Keywords:** colorectal cancer, gene expression profiling, molecular stratification, biopsy, transcriptional signatures, intrinsic subtypes, consensus molecular subtypes

## Abstract

Colorectal cancer (CRC) biopsies underpin accurate diagnosis, but are also relevant for patient stratification in molecularly‐guided clinical trials. The consensus molecular subtypes (CMSs) and colorectal cancer intrinsic subtypes (CRISs) transcriptional signatures have potential clinical utility for improving prognostic/predictive patient assignment. However, their ability to provide robust classification, particularly in pretreatment biopsies from multiple regions or at different time points, remains untested. In this study, we undertook a comprehensive assessment of the robustness of CRC transcriptional signatures, including CRIS and CMS, using a range of tumour sampling methodologies currently employed in clinical and translational research. These include analyses using (i) laser‐capture microdissected CRC tissue, (ii) eight publically available rectal cancer biopsy data sets (n = 543), (iii) serial biopsies (from AXEBeam trial, NCT00828672; n = 10), (iv) multi‐regional biopsies from colon tumours (n = 29 biopsies, n = 7 tumours), and (v) pretreatment biopsies from the phase II rectal cancer trial COPERNCIUS (NCT01263171; n = 44). Compared to previous results obtained using CRC resection material, we demonstrate that CMS classification in biopsy tissue is significantly less capable of reliably classifying patient subtype (43% unknown in biopsy versus 13% unknown in resections, p = 0.0001). In contrast, there was no significant difference in classification rate between biopsies and resections when using the CRIS classifier. Additionally, we demonstrated that CRIS provides significantly better spatially‐ and temporally‐ robust classification of molecular subtypes in CRC primary tumour tissue compared to CMS (p = 0.003 and p = 0.02, respectively). These findings have potential to inform ongoing biopsy‐based patient stratification in CRC, enabling robust and stable assignment of patients into clinically‐informative arms of prospective multi‐arm, multi‐stage clinical trials. © 2018 The Authors. *The Journal of Pathology* published by John Wiley & Sons Ltd on behalf of Pathological Society of Great Britain and Ireland.

## Introduction

Recent studies have defined the molecular taxonomy of colorectal cancer (CRC) by transcriptional, methylation, and mutational profiling 1, 2, 3, 4, 5, culminating in the publication of four consensus molecular subtypes (CMSs) 6, two of which reflect pathological well‐defined entities within the tumour microenvironment (TME): CMS1 (high immune‐cell infiltration; better prognosis) and CMS4 (high relative density of stroma, particularly fibroblasts; poorer prognosis) 7. A second classification, the CRC intrinsic subtypes (CRISs), utilises epithelial‐specific gene expression to potentially provide prognostic/predictive value 8, 9.

Using macrodissected tissue from the central tumour (CT), invasive front (IF), and lymph node (LN) from individual patients (patients, *n =* 24; samples, *n =* 72), we previously demonstrated the potential for discordant assignment of these patient‐of‐origin matched samples when using transcriptional classifiers, as different CMS classifications were mapped to different regions of the same tumour, due to stromal‐derived intratumoural heterogeneity (ITH) 10. We further demonstrated that this confounding effect could be resolved by using epithelial‐rich or cancer‐cell intrinsic subtypes, such as CRISs, which demonstrated superior ‘spatial concordance’, with identical CRIS classification achieved across multiple regions‐of‐origin in patient‐matched samples 11.

The potential clinical utility of both CMS and CRIS molecular subtyping has been extensively validated in CRC **resection** specimens, and while molecular profiling of surgical resection material is possible in large retrospective studies 1, the suitability of CRC **biopsy** material for prospective molecular stratification has not been comprehensively assessed. This is increasingly important, given the number of molecularly‐guided CRC trials that require profiling of pretreatment biopsies for patient stratification 12.

In this study, we assessed the spatial and temporal stability of clinically‐relevant molecular signatures in diagnostic biopsy material in three potentially clinically‐relevant scenarios. We utilised a multi‐regional (CT and IF) laser capture‐microdissected (LCM) CRC cohort to examine if stromal ITH occurs with this more precise specimen‐preparation methodology. Additionally, we assessed subtyping robustness in a meta‐analysis of publicly available rectal cancer biopsy datasets. We also performed temporal/spatial assessment of the stability of these classifiers, using both patient‐matched serial biopsies collected over a 3‐week period from the phase II AXEBeam study 13 and multi‐region‐of‐origin colon biopsies from the Biopsies of Surgical Specimens (BOSS) study 14. Finally, as part of the S:CORT (Stratification in COloRecTal cancer) research programme 12, 15, we assessed the ability of CRISs and CMSs to classify histologically‐diverse rectal biopsy samples from the phase II COPERNICUS study.

## Materials and methods

### Study design

The study design is summarised in the supplementary material, Figure S1, with details of the patient cohorts outlined below. Initially, we assessed the patient‐clustering capabilities of CRC gene signatures in an LCM cohort of invasive front (IF) and central tumour (CT) regions. We assessed the proportions of CRIS and CMS molecular subtypes 6, 8 in biopsy material from publically available rectal cancer biopsy gene expression datasets (in GEO); the details of these cohorts are outlined in Table 1. We assessed the temporal and spatial stability of CRIS and CMS signatures in biopsy samples from AXEBeam and BOSS studies, respectively 13, 14. Finally, we performed molecular analysis of biopsies from the COPERNICUS study.

**Table 1:** The eight rectal cancer biopsy gene expression datasets curated from GEO, their sample size, and the gene expression profiling platform used

Dataset	Sample size	Platform
GSE56699	58	Illumina WG‐DASL
GSE94104	48	Illumina WG‐DASL
GSE3493	46	Affymetrix Human Genome U95 Version 2 Array
GSE68204	38	Agilent‐014850 Whole Genome Microarray 4×44K
GSE35452	46	Affymetrix Human Genome U133 Plus 2.0 Array
GSE46862	69	Affymetrix Human Gene 1.0 ST Array
GSE45404	42	Affymetrix Human Genome U133 Plus 2.0 Array
GSE87211	196	Agilent‐014850 Whole Genome Microarray 4×44K
Total	543	

### Publically available datasets

All public datasets were downloaded from the gene expression omnibus (GEO) (https://www.ncbi.nlm.nih.gov/geo). Rectal cancer biopsy datasets are detailed in Table 1. All datasets with sufficient probe‐to‐gene annotations and sample size (*n* > 20) were curated. When possible, raw unprocessed data were downloaded and expression profiles underwent standard Robust Multi‐array Average (RMA) normalisation prior to molecular subtyping. When only post‐processed data were available, we downloaded series matrices to perform molecular subtyping. All probes were used and no variance filtering was performed on any data prior to molecular subtyping, to ensure the presence of all 273 CMS genes and 565 CRIS genes from the published classification models.

### LCM CRC cohort

GSE65480 is composed of LCM CRC tissue from 20 matched IF and CT regions, profiled using the Affymetrix Human Gene 1.0 ST Array.

### Colon and rectal cancer biopsy datasets

Full details for the arrays employed and sample numbers analysed for each cohort are detailed in Table 1, with GEO accession numbers and brief clinical details for the rectal cancer meta‐dataset summarised below. GSE56699 consists of 58 pretreatment rectal cancer formalin‐fixed, paraffin‐embedded (FFPE) biopsy specimens from patients treated with preoperative radiotherapy. GSE94104 consists of 48 locally advanced rectal cancer (LARC) pretreatment biopsy specimens from patients treated with long‐course preoperative 5‐fluorouracil (5‐FU)‐based chemoradiotherapy. GSE3493 contains 46 pretreatment rectal cancer biopsies from patients treated with preoperative radiation. GSE68204 comprises 38 pretreatment LARC biopsy specimens from patients treated with preoperative chemoradiotherapy. GSE35452 consists of 46 pretreatment rectal cancer biopsies from patients treated with 5‐FU‐ and irinotecan‐based preoperative chemoradiotherapy. GSE46862 contains 69 rectal cancer pretreatment biopsies from patients treated with preoperative chemoradiotherapy. GSE45404 consists of 42 pretreatment rectal cancer biopsies from patients treated with preoperative 5‐FU and oxaliplatin‐based preoperative chemoradiotherapy. We utilised 196 pretreatment rectal cancer biopsies from GSE87211, where patients were treated with a preoperative chemoradiotherapy regimen consisting of 5‐FU alone and FOLFOX.

### Colon cancer multi‐region‐of‐origin biopsy cohort

To assess the spatial stability of CMSs and CRISs in biopsy samples, we utilised transcriptional profiles from the BOSS study, which were downloaded from GSE85043. This dataset consists of 29 multi‐regional biopsies from seven patients. Samples were profiled using the Affymetrix Human Genome U133 Plus 2.0 Array. Importantly, each biopsy had been randomly taken from the surgical specimen using endoscopic biopsy forceps to simulate the clinical environment.

### Longitudinal serial rectal biopsy cohort

Material from ten matched biopsy samples from patients recruited to the AXEBeam phase II trial (NCT00828672; GSE60331) was profiled using the Affymetrix Primeview array. This trial investigated the efficacy of bevacizumab/chemo‐radiation combination in rectal cancer. Biopsies were taken before therapy and 3 weeks into the first cycle of bevacizumab, but before chemo‐radiation.

### Clinical trial cohort

COPERNICUS is a phase II study of neoadjuvant oxaliplatin‐based chemotherapy, followed by short‐course radiotherapy and surgical resection in patients with rectal cancer. Within S:CORT, we generated transcriptional profiles using the Affymetrix Almac Xcel array from the COPERNICUS (NCT01263171) trial cohort (52 biopsy samples); 50 samples (96.2%) generated suitable quantities of RNA for analysis, while 44 (84.6%) yielded robust transcriptional profiles.

### Gene signatures

We previously evaluated eight CRC gene expression signatures for variation in their ability to robustly cluster matched multi‐region‐of‐origin CRC gene expression profiles 11. To validate the novel results generated in the current study using an independent dataset, we employed the same eight gene expression signatures as previously published 11. The 30‐gene signature was developed as a classifier of ‘region‐of‐origin’ from a cohort of 24 patient samples using patient‐matched samples from IF, CT, and LN regions (total *n =* 24). This cohort is available from the NCBI GEO repository under accession number GSE95109. 10 The Jorissen *et al* signature 16 was developed using transcriptional profiles from 553 colorectal samples using Affymetrix Human Genome U133 Plus 2.0 Arrays, to develop a 163‐gene ‘metastasis classifier’, which could stratify stage B and C samples into prognostic subtypes. The Eschrich *et al* signature 17 was developed using cDNA array profiles from 78 colon tumour samples to generate a 43‐gene prognostic signature. The Sadanandam *et al* signature 5 (a surrogate for CMS) was developed using transcriptional profiles from 445 primary CRC resections using Affymetrix HG‐U133Plus2.0 GeneChip arrays to define 786 subtype‐specific signature genes. The 207 genes associated with classification of the ‘stem‐like’ subtype from the original Sadanandam *et al* signature were used as our stem‐like (CMS4) signature. The Kennedy *et al* signature 18 used stage II FFPE colon cancer tumours on the Almac Colorectal Cancer DSA platform to define a 634‐probeset stage II prognostic signature. The Popovici *et al* signature 19 was developed using 668 stage II/III FFPE colon cancer tissue samples from the PETACC‐3 phase III clinical trial on the Almac Colorectal Cancer DSA platform. A 64‐gene classifier was developed which identified samples with signalling similar to *BRAF*‐mutant tumours. The colorectal intrinsic signature (CRIS) 9 was developed using transcriptional profiles from 515 patient‐derived xenograft tumours using Illumina human‐specific 48 k gene chips. A 565‐gene classifier was developed which identified five subtypes based on their intrinsic epithelial expression profile.

We previously indicated that the 30‐gene, stem‐like (CMS4) Jorissen and Eschrich gene signatures contain genes highly expressed in fibroblasts; the Sadanandam (CMS) and Kennedy signatures have a more balanced expression across cell types, whilst the Popovici and CRIS gene signatures contain predominantly epithelial‐specific gene signatures 11.

In addition to the previous eight gene expression signatures, we assessed the clustering capabilities of a recently published refined CMS protein expression classifier 20, which consists of four proteins – CDX2, FRMD6, HTR2B, and ZEB1*,* developed using tissue microarray analysis in combination with MSI genotyping, to classify CMS1 (based solely on MSI) and combined CMS2/3 and CMS4 subtypes. In this publication, CDX2 is used as a marker for epithelial‐like tumours (CMS2/3), whereas FRMD6, ZEB1, and HTR2B have higher expression in mesenchymal‐like tumours (CMS4).

### Patient classification

To validate the improved ability of CRIS gene signatures to classify by patient‐of‐origin rather than region‐of‐origin, we utilised divisive analysis clustering (DIANA) and normalised Pearson similarity scoring. This Pearson score was used to define the ratio between the covariance and the standard deviation of the multi‐region CRC samples, where higher ratios (up to 1) indicate increased similarity. These two methodologies, as previously published, assess variation in clustering between gene signatures 11. CMS and CRIS subtypes were assigned to each gene expression profile using the previously published methods 6, 8, 11. This combined approach of utilising the published molecular subtyping CMS and CRIS classifiers, alongside two independent patient clustering methods (Pearson similarity score and DIANA), will reduce the possibility that our findings are confounded by a methodology bias specific to any particular classification algorithm.

### Statistical analysis and graphical representation

Other statistical analyses, including Fisher's exact and unpaired *t*‐tests, were performed using GraphPad Prism 6 (GraphPad Software, La Jolla, CA, USA). Plots for integrative visualisation purposes were generated using StratomeX tool within Caleydo software version 3.1.5 downloaded from http://caleydo.org/tools/stratomex.

### Assessing tumour content in COPERNICUS samples

A visual assessment of neoplastic cell content, performed only within the macrodissected area of tissue used for molecular profiling, was made at 4× magnification. This value was estimated by a pathologist blinded to CMS and molecular data.

## Results

### Patient stratification in epithelial‐enriched LCM CRC specimens

Samples from a cohort of CRC tumour resection tissue samples that had been dissected into CT and IF regions using LCM were evaluated using a series of transcriptional profiling approaches (see the Materials and methods section). Importantly, as this dataset has been generated using LCM epithelial tissue, it more closely resembles an epithelial‐enriched CRC biopsy sample, rather than the macrodissected resection tissue used in our previous studies 10, 11.

**Figure 1:**
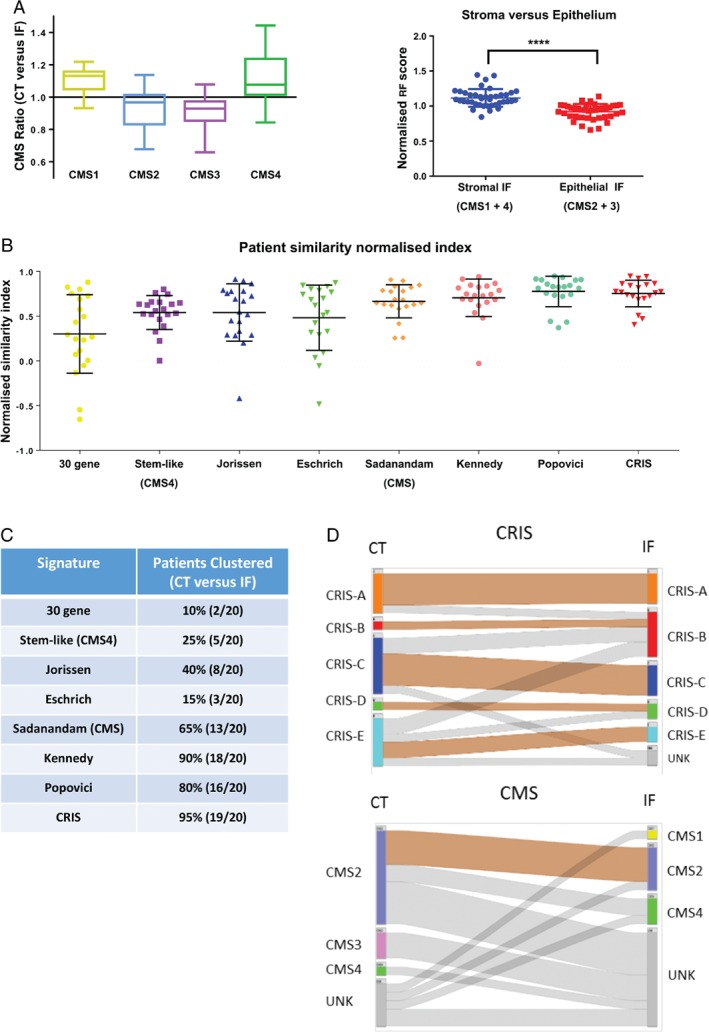
Patient stratification using CRC cell intrinsic signatures. (A) Left: using the CMS classifier, each sample will be assigned an individual score for CMS1, CMS2, CMS3, and CMS4. Box plots showing the relative CMS ratio for CMS1–4 in patient‐matched central tumour (CT) and invasive front (IF) samples (n = 20). Right: dot plot comparing normalised random forest posterior probability scores for IF front region of stromal and epithelial CMS subtypes (p = 0.0001, Student's t‐test). (B) Dot plot of normalised Pearson similarity scores for each gene signature. (C) Table showing clustering concordance by gene signature. (D) Caleydo (Stratomex) integrative visualisation of CRIS and CMS concordance between matched CT and IF regions.

First, we employed the published CMS classifier [which uses a random forest (RF) posterior probability score] to evaluate each matched CT and IF sample. Using this method, each tissue sample is assigned a score for each individual CMS class (i.e. a sample will have a score for CMS1, CMS2, CMS3, and CMS4) before a final classification is made. Using these individual CMS scores, we created a CMS ratio based on the change in RF score, from CT and IF regions, for each patient‐matched sample (Figure 1A). We demonstrated an increase in the relative classification score for CMS1 and 4 subtypes (the stromal subtypes) in IF samples compared with patient‐matched CT samples in this LCM cohort. In contrast, the ratios for CMS2 and 3 (the epithelial subtypes) showed a decrease in the IF regions compared with the CT regions (Figure 1A, left, GSE65480, *n =* 20; and supplementary material, Figure S2A). When comparing the normalised RF classification scores between combined stromal (CMS1, 4) and epithelial subtypes (CMS2, 3), we observed a statistically significant difference between the two groups (Figure 1A, right, Student's *t*‐test, *p =* 0.0001; and supplementary material, Figure S2B).

We next used a Pearson similarity score in conjunction with eight CRC‐specific classifiers (see the Materials and methods section and ref 11) to assess the robustness of classification in these patient‐matched samples. This Pearson similarity analysis indicates variation in transcriptional classification of patient‐matched samples (higher ratios indicate increased similarity), allowing a focus on the biology underlying the classification system. Using this method, we highlighted high levels of concordance of patient‐matched samples from different regions of the tumour when using gene signatures that focused on cancer cell intrinsic signalling (CRIS, Popovici) compared with stromal‐dependent signatures (Figure 1B). Divisive clustering, using the DIANA methodology, also demonstrated that these signatures correctly clustered patient‐matched samples from different tumour regions (CRIS 95%, Popovici 85%) compared with stromal‐derived signatures (Figure 1C and supplementary material, Figure S3). Furthermore, we attempted to use our transcriptional data in combination with a refined CMS classifier 20, with the caveat that this refined classifier was originally developed using four protein expression immunohistochemistry (IHC) markers to distinguish CMS2/3 from CMS4. Using this refined IHC CMS classifier, we again observed a poor patient‐matching correlation of our transcriptional data using DIANA (supplementary material, Figure S4).

Additionally, when re‐employing the CMS RF classifier, alongside the nearest template predictor (NTP) CRIS classification method, we observed increased concordance in spatial stability (correct identification of patient‐of‐origin) in multi‐regional samples when employing CRIS as opposed to CMS classification (Figure 1D; CRIS concordance 60% versus CMS 15%, *p =* 0.003, Fisher's exact). We observed that 40% of all LCM cohort samples profiled could not be confidently assigned to a CMS group (termed UNK), particularly in IF samples; only 5% CRIS‐UNKs are observed in the same sample series (Figure 1D; *p =* 0.0001, Fisher's exact).

### Molecular subtype assessment in CRC biopsy meta‐dataset

We utilised the online repository GEO by searching for ‘rectal cancer’ datasets (to 1 March 2017) to curate a meta‐dataset containing 543 treatment‐naïve rectal cancer biopsy gene expression profiles from eight independent datasets (full details in the Materials and methods section and Table 1). This meta‐dataset consists of gene expression profiles from five different gene expression platforms, enabling both comparative assessment between molecular subtyping techniques and cross‐platform correlation (Table 1).

**Figure 2:**
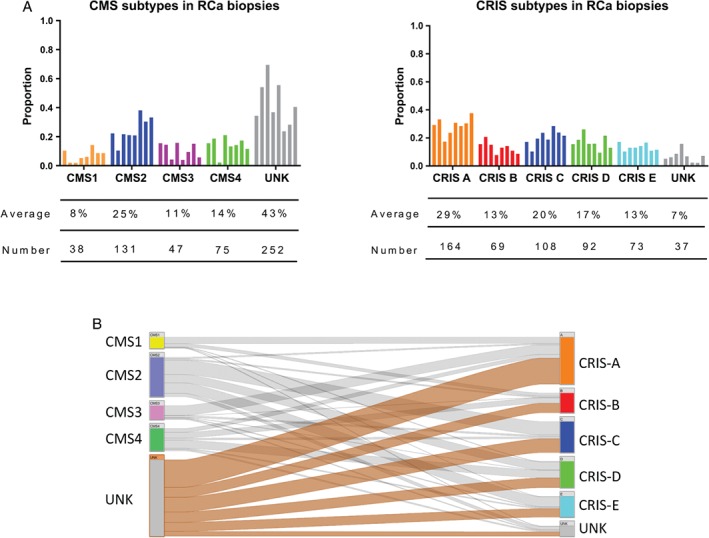
Molecular subtyping of rectal cancer biopsies. (A) Bar charts showing the proportions, average, and total numbers of each CMS and CRIS group across the eight rectal cancer biopsy datasets. (B) Caleydo (Stratomex) integrative visualisation of CMS and CRIS across the eight rectal cancer biopsy datasets.

We classified each individual dataset using the CMS method 6, resulting in the assignment of a UNK classification in 43% (*n =* 252) of patient samples (Figure 2A, B; range 24–70%). This finding, specifically in biopsy samples, is considerably higher than the 13% previously observed in CRC resections by Guinney *et al* (*p =* 0.0001, Fisher's exact) 6. In contrast, CRIS classification in the same datasets revealed that only 7% (*n =* 37) of patients were UNK (Figure 2A, B; range 2–16%). This observed proportion of CRIS‐UNKs across these biopsy datasets correlates with the 9.2% CRIS‐UNKs identified by Isella *et al* 8 in CRC resection specimens (342 UNKs from a total of 3738 samples) (no significant difference, *p =* 0.07, Fisher's exact). Direct comparison of CMS and CRIS classifications for each of the 543 rectal cancer biopsies revealed that 94% of CMS‐UNK patients could subsequently be assigned a CRIS subclass (Figure 1C), indicating that the transcriptomics data are of sufficient quality for reliable classification. From these results, in addition to the previously identified confounding issues with stromal‐derived ITH when using the CMS classifier, we have demonstrated for the first time CRC patients when using pretreatment biopsy samples.

### Temporal stability of molecular subtypes in serial biopsy samples

**Figure 3:**
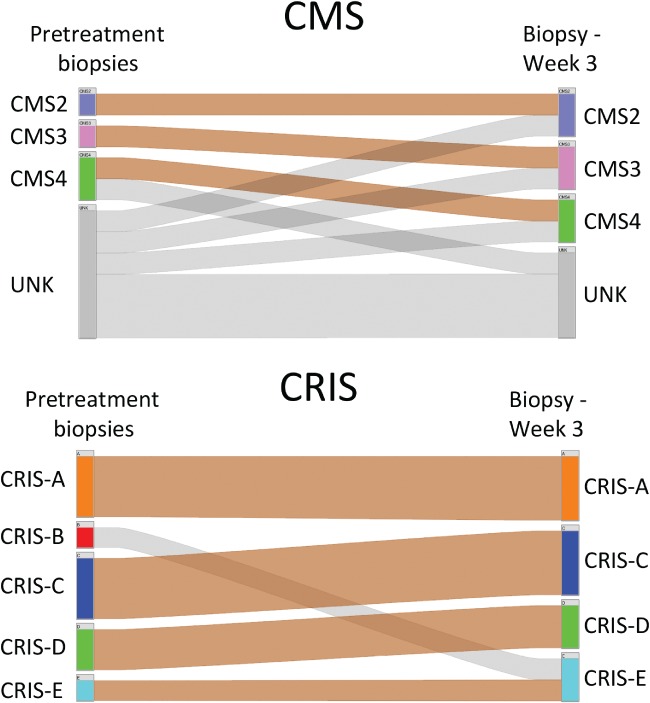
Temporal stability of molecular subtypes in serial biopsies. Caleydo (Stratomex) integrative visualisation of CRIS and CMS concordance in serial rectal cancer biopsies from the AXEBeam trial (n = 10).

Serial biopsies can provide information on treatment response and clinically‐relevant changes in tumour biology; therefore, evaluating the temporal stability of molecular subtypes in repeat CRC biopsies is highly relevant. We analysed the transcriptional profiles of ten patient‐matched serial biopsy samples (taken both before and following 3 weeks of bevacizumab treatment) from the AXEBeam phase II trial (NCT00828672; GSE60331). Again, we confirmed a high number of UNK samples by CMS analysis (50%, 10/20), with only 30% (3/10) of patients displaying a concordant CMS classification; lack of classification does not appear to be due to treatment‐induced transcriptional changes, as six of the ten UNK samples were obtained before treatment. Conversely, all samples were classified by CRIS, with 90% (9/10) temporal concordance across matched serial biopsies taken during this clinical trial (Figure 3, p = 0.02, Fisher's exact).

### Spatial stability of molecular subtypes in biopsies of surgical specimens

**Figure 4:**
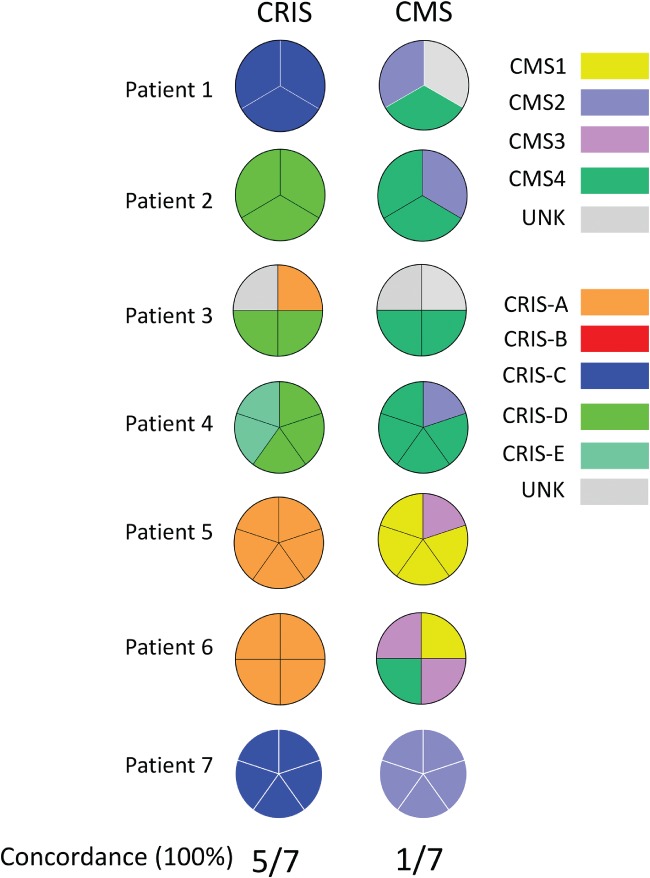
Spatial stability of molecular subtypes in multi‐regional biopsies. Pie charts showing the concordant classification of multi‐regional biopsies from seven surgical specimens in the BOSS study into CRIS (left) and CMS (right) subtypes.

We have demonstrated that the CRIS classifier provides a more spatially robust classification than CMS in multi‐region‐of‐origin LCM CRC cells (Figure 1D). However, pretreatment biopsies, rather than resection tissue, are increasingly being used for prospective molecular stratification. Therefore, we subtyped 29 multi‐regional biopsies originating from seven CRC surgical specimens (between three and five multiple regions‐of‐origin samples per patient) from the BOSS study (GSE85043), using CMS and CRIS classifiers. We demonstrated that only 1/7 tumours subtyped had 100% concordance in all regions biopsied using the CMS classifier, whereas 5/7 tumours had 100% concordance using the CRIS classifier (Figure 4). Despite the small sample size in this cohort, these findings further confirm our observations from the LCM CRC cohort (Figure 1D) that CRIS shows greater spatial stability than CMS in clinically‐relevant biopsy material.

### Patient stratification in prospective clinical trial biopsy material

**Figure 5:**
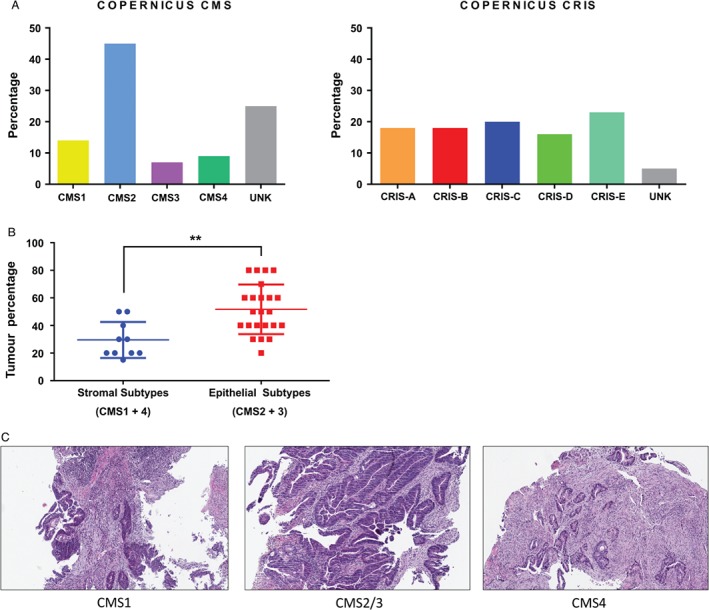
Molecular subtyping and tumour content in biopsy material from the phase II COPERNICUS clinical trial. (A) Bar charts showing the percentage of patients from each subtype, CMS (left) and CRIS (right), in the COPERNICUS cohort. (B) Dot plots comparing the tumour percentage between stromal subtypes (CMS1 and 4) and epithelial subtypes (CMS2 and 3) (Student's t‐test, p = 0.003). (C) Representative H&E images of CMS1 (left), CMS2/3 (middle), and CMS4 (right) biopsies (×10 original magnification).

Using transcriptional profiles from COPERNICUS (n = 44) (see the Materials and methods section), generated within S:CORT 12, we observed a higher percentage of patients classified as UNKs when using CMS compared with CRIS (Figure 5A; 25% versus 5%, p = 0.013, Fisher's exact). A detailed pathological review of haematoxylin and eosin (H&E) specimens was performed to test the ability of histological feature assessment to predict CMS subtypes, particularly for the CMS1/CMS4 stromal‐dependent subtypes. In a masked pathological analysis, we observed that a lower tumour and higher stromal percentage correlated with increased CMS1/CMS4 classification scores (Figure 5B, p = 0.003, Student's t‐test), again emphasising the histopathological features underlying this classification system. This is depicted in Figure 5C by the representative H&E images of CMS1 (immune‐enriched), CMS2/3 (epithelial‐enriched), and CMS4 (fibroblast‐enriched) biopsies.

## Discussion

Transcriptomic dissection of CRC tumours has identified two molecular classifiers with potential clinical relevance. The CMS classifier identifies two histological subtypes – CMS1 (immune‐rich) and CMS4 (stromal‐rich) – and two epithelial‐rich subtypes – CMS2 (upregulated for WNT and MYC pathways) and CMS3 (enriched for *KRAS* mutations and activation of metabolic pathways). In contrast, the CRIS classifier identifies five tumour subtypes based on cancer cell intrinsic biology from within the TME. In this study, we assessed for the first time the ability of the CMS and CRIS molecular subtypes to robustly classify tumour samples, with particular emphasis on prospective pretreatment biopsy tissue, when confronted with potential spatial and/or temporal confounders. Initially, using a cohort of patient‐matched LCM invasive front and central tumour regions from CRC resections, we demonstrated that the epithelial enrichment achieved by LCM is not sufficient to overcome the confounding effect of stromal intratumour heterogeneity. These results validate our previous findings that CRIS is a more robust patient stratifier than CMS, while also indicating that epithelial enrichment using the precise but time‐consuming LCM method cannot eliminate the potential for stromal‐derived ITH to undermine patient stratification. Our assessment of 543 rectal cancer biopsies (the largest rectal cancer dataset compiled to date) also revealed a significantly larger proportion of unclassified biopsies than has previously been reported for resection samples when using the CMS classifier. In contrast, the CRIS classifier assigned the same biopsies into proportions consistent with those observed in resection material. This observation indicates that while CMS classification provides important prognostic information in CRC **resection** samples, it may not be suited to classification in FFPE **biopsy** material.

We also demonstrated increased temporal concordance with the CRIS classifier when assessing longitudinal rectal cancer biopsies from patients recruited to the phase II AXEBeam clinical trial. As temporal stability of molecular subtypes could be confounded by therapy‐related gene expression alteration 21 (although this is not indicated by our current analysis), we believe that this observation warrants further investigation in treatment‐naïve samples or indeed with standard‐of‐care chemotherapy samples, in order to fully understand the implications of this evolving biology. In line with our analysis in the LCM CRC cohort and the rectal cancer meta‐dataset, we again highlighted the superior spatial stability of CRIS compared with CMS in a multiple region‐of‐origin cohort using colon cancer biopsies (BOSS study). Finally, we coupled histopathological assessment and molecular subtyping of pretreatment rectal cancer biopsies from the phase II COPERNICUS clinical trial, where we observed low tumour percentage (and high stromal content) to be correlated with the stromal CMS subtypes (CMS1 and 4).

**Figure 6:**
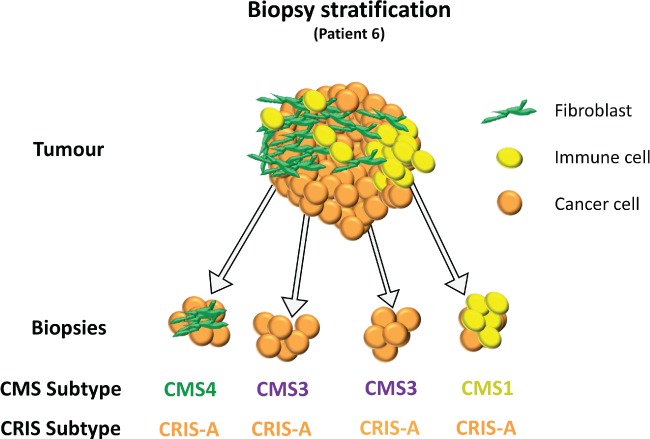
Proposed model of stromal heterogeneity confounding CMS subtyping in colorectal cancer biopsies depicting the molecular classification for Patient 6 from the BOSS analysis in Figure 4.

CRC biopsies are currently used for both cancer diagnosis and patient stratification, employing small panels of clinically important biomarkers, such as *RAS* mutational status, although despite providing useful clinical information, they currently lack both prognostic and positive predictive value. Increasingly, biopsy samples are being considered for molecular stratification using high‐throughput transcriptional profiling, particularly in the adjuvant/neoadjuvant clinical trial setting, to aid in patient assignment into prognostic and/or predictive subgroups. The prognostic and predictive potential of CMS (and CRIS) molecular subtypes has, to date, been investigated using large retrospective collections of resected CRC tissue 6, 8; our present study highlights the need for rigorous testing and refinement of CRC classifiers using prospective biopsy tissue, thus facilitating their employment as clinically‐useful tools in patient stratification. Molecular analysis of patient 6 from the BOSS study (see Figure 4) illustrates the point; all four biopsy samples from across the surgical specimen were assigned CRIS‐A classification (100% concordance), whereas multiple CMS classifications were assigned from the same four biopsy samples, including CMS3 (2/4 biopsies), CMS1 (1/4 biopsies), and CMS4 (1/4 biopsies). Given the current prognostic algorithm associated with CMS classification, these results would be of little utility in patient stratification, as they would reveal a patient who has a tumour with either a good prognosis (CMS1), an intermediate prognosis (CMS3) or a poor prognosis (CMS4), depending on the region of origin of the biopsy sample. The 100% concordance observed with CRIS classification, independent of region of origin, suggests that CRIS classification is the methodology of choice when using a single biopsy approach to patient stratification. Ubink *et al* indicate via their analysis that setting a threshold for CMS4 detection across multiple biopsies may help to ensure a more robust classification 14. However, taking multiple biopsies across the IF and CT regions of a tumour in the clinical setting may not always be feasible, nor is it part of current standard pathology practice. In contrast to the robust and reproducible nature of CMS classification in large resection tissue samples 6, our data reveal multiple conflicting subtype assignments, depending on the tumoural region sampled during tissue collection, with stromal‐based classifiers like CMS specifically when using biopsy samples. We propose that using the CRIS classifier transcends this stromal heterogeneity, resulting in a robust patient classification methodology regardless of the proportions of TME‐derived material even in biopsy tissue (Figure 6).

Biomarker‐informed clinical trials such as FOxTROT (ISRCTN 87163246) and FOCUS4 (ISRCTN90061546) have involved the application of multiple molecular tests on biopsy material, which may be limited in quantity (and potentially quality), following diagnostic assessment. While these studies have employed mutational status for patient stratification, evaluation of transcriptional‐based signatures in collaborative programmes such as S:CORT aims to provide a clinical rationale for such stratification in clinical practice. There is no doubt as to the potential clinical importance of the TME and CMS classification system, with numerous studies highlighting its prognostic value. However, given the nature of stromal ITH and the current lack of a standardised method for the collection of biopsy material, even within ongoing clinical trials, this method can easily be confounded by sampling bias. The implementation of a standardised biopsy collection method may remove this confounding issue, but until such a reproducible biopsy protocol is developed, our data support the use of CRIS stratification as the molecular pathology methodology of choice underpinning reproducible prospective patient stratification from current routine biopsy tissue.

In addition to a robust subtype assignment and clear prognostic value, the clinical relevance of defining CRIS lies in its potential predictive value, which gives insights into the biology underlying the epithelial component of the tumour, which may in turn guide an informed (targeted) therapy approach. We have previously shown that CRIS‐C patient‐derived xenografts (PDX) respond to EGFR inhibition (cetuximab) 8, which was further validated using tumour profiles from a phase II metastatic CRC study 22. Preliminary results from FOxTROT have confirmed the feasibility of stratifying colon cancer patients, using pretreatment biopsies, for targeted (panitumumab) and/or cytotoxic chemotherapy treatment in the neoadjuvant setting. The data presented here support the use of CRIS profiling of pretreatment biopsy material to inform precision oncology stratification based on the specific biology of the disease, determined using diagnostic endoscopic tissue. The ‘window‐of‐opportunity’ study design, as used in FOxTROT, urgently requires robust biomarkers linked to distinct therapeutic choices in order to select patients for more personalised treatments. Based on our findings, classification of samples based on cancer‐cell intrinsic properties, such as CRIS, is necessary to guide testing of novel treatment interventions in the first‐line preoperative setting, where they have the greatest chance of achieving therapeutic response(s).

In conclusion, we highlight the robust nature of the CRIS transcriptional classifier in diagnostic endoscopic biopsy material, which is the relevant entry point to ongoing and forthcoming CRC clinical trials. The limitations of CMS identified previously by our group are still evident when using LCM processing of samples, suggesting that this time‐consuming method does not eliminate the potential for ITH to confound patient classification, as previously identified in macrodissected samples. Given the limited control over the spatial region‐of‐origin of biopsy tissue available for analysis, our current data support patient stratification using CRIS transcriptional subtypes, which minimise potentially confounding ITH. This work provides a strong rationale to investigate the prognostic/predictive value of CRIS subtypes in biopsy‐led and statistically‐powered prospective CRC trials.

## Author contributions statement

All authors wrote and critiqued the manuscript. MA, SDR, PS, AMBM, AR, CI, EM, DGM, ML, and PDD generated, analysed and interpreted data. SG, CH, RA, DV, TM, ML, and PDD were the clinical trial and SCORT Management Group; MA, ML, and PDD designed the study.

**SUPPLEMENTARY MATERIAL ONLINE**

**Supplementary figure legends**

**Figure S1.** Study design

**Figure S2.** Comparison of normalised random forest scores between stromal subtypes (CMS1 and 4) and epithelial subtypes (CMS2 and 3)

**Figure S3.** Assessment of divisive clustering capabilities in matched CRC CT and IF regions using eight previously published CRC gene expression signatures

**Figure S4.** Assessment of the clustering capabilities of the refined CMS classifier published by Trinh *et al* 20

## Supporting information


**Supplementary figure legends**

**Figure S1.** Study design.

**Figure S2.** Comparison of normalised random forest scores between stromal subtypes (CMS1 and 4) and epithelial subtypes (CMS2 and 3). (A) Line plots showing changes in normalised random forest score between CT and IF for CMS1–4 (B) Left: line plot of normalised stromal CMS random forest scores between CT and IF. Right: line plot of normalised epithelial CMS random forest scores between CT and IF.

**Figure S3.** Assessment of divisive clustering capabilities in matched CRC CT and IF regions using eight previously published CRC gene expression signatures. (A) 30 gene; (B) Sadanandam 5; (C) Eschrich 17; (D) stem‐like (CMS4); (E) Jorissen 16; (F) Kennedy 18; (G) Popovici 19; and (H) CRIS 9.

**Figure S4.** Assessment of the clustering capabilities of the refined CMS classifier published by Trinh et al. 20. Divisive analysis clustering in matched CRC CT and IF regions using the Trinh gene expression signature.
