# Supplementary material for: Prospective patient stratification into robust cancer‐cell intrinsic subtypes from colorectal cancer biopsies
Source: J Pathol. 2018 Mar 25;245(1):19–28. doi: 10.1002/path.5051 (PMC5947827; doi:10.1002/path.5051)
Supplement: Supplementary file 1 — Supplementary figure legends [file PATH-245-19-s005.docx]

**Supplementary figure legends**

**Figure S1.** Study design.

**Figure S2.** Comparison of normalised random forest scores between stromal subtypes (CMS1 and 4) and epithelial subtypes (CMS2 and 3). (A) Line plots showing changes in normalised random forest score between CT and IF for CMS1–4 (B) Left: line plot of normalised stromal CMS random forest scores between CT and IF. Right: line plot of normalised epithelial CMS random forest scores between CT and IF.

**Figure S3.** Assessment of divisive clustering capabilities in matched CRC CT and IF regions using eight previously published CRC gene expression signatures. (A) 30 gene; (B) Sadanandam [5]; (C) Eschrich [17]; (D) stem-like (CMS4) [5]; (E) Jorissen [16]; (F) Kennedy [18]; (G) Popovici [19]; and (H) CRIS [9].

**Figure S4.** Assessment of the clustering capabilities of the refined CMS classifier published by Trinh *et al* [20]. Divisive analysis clustering in matched CRC CT and IF regions using the Trinh gene expression signature.
