# Supplementary figures and images for: Prospective patient stratification into robust cancer‐cell intrinsic subtypes from colorectal cancer biopsies
Source: J Pathol. 2018 Mar 25;245(1):19–28. doi: 10.1002/path.5051 (PMC5947827; doi:10.1002/path.5051)

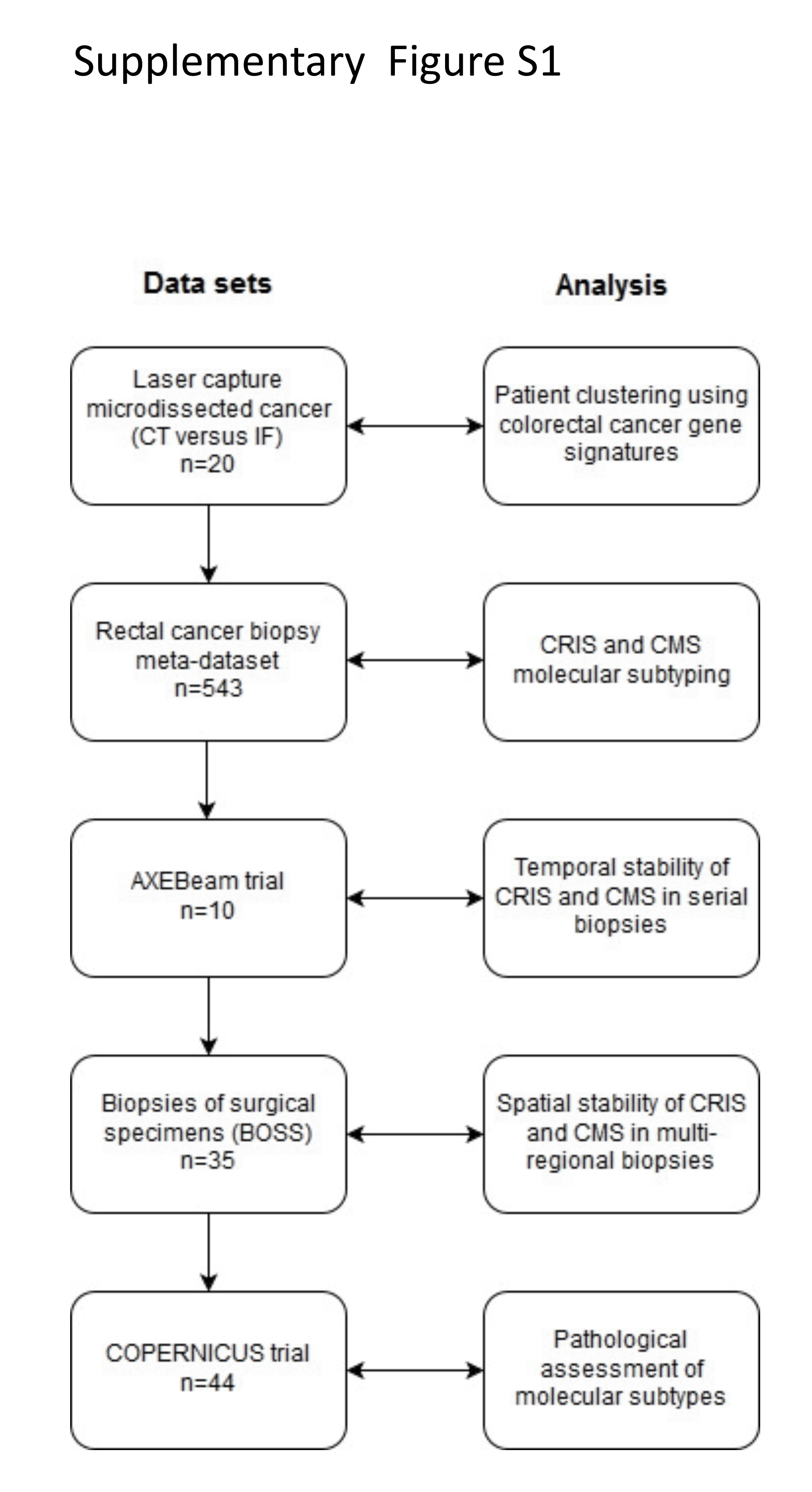

Supplement: Supplementary file 2 — Figure S1. Study design. [file PATH-245-19-s001.tif]

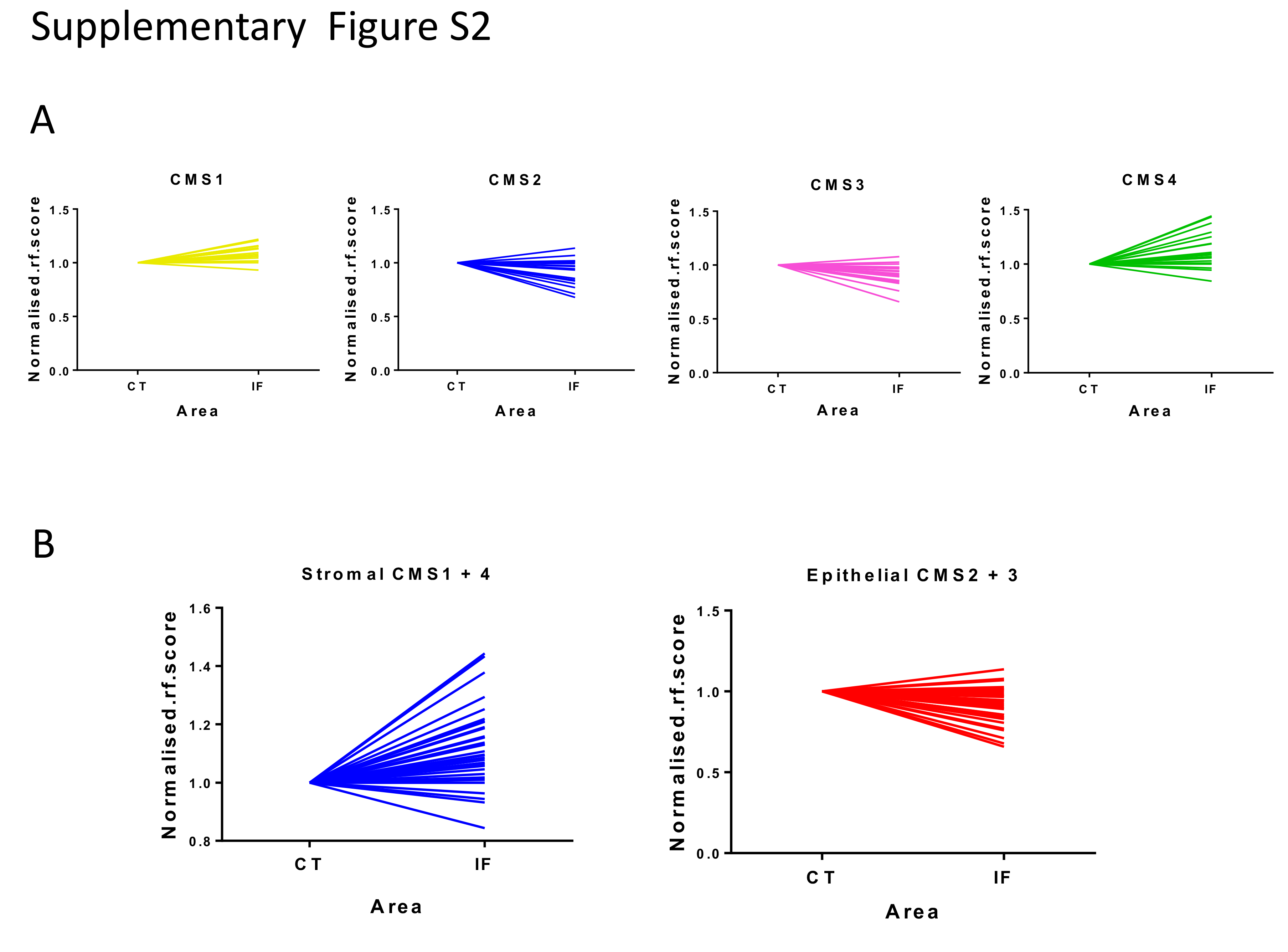

Supplement: Supplementary file 3 — Figure S2. Comparison of normalised random forest scores between stromal subtypes (CMS1 and 4) and epithelial subtypes (CMS2 and 3). (A) Line plots showing changes in normalised random forest score between CT and IF for CMS1–4 (B) Left: line plot of normalised stromal CMS random forest scores between CT and IF. Right: line plot of normalised epithelial CMS random forest scores between CT and IF. [file PATH-245-19-s002.tif]

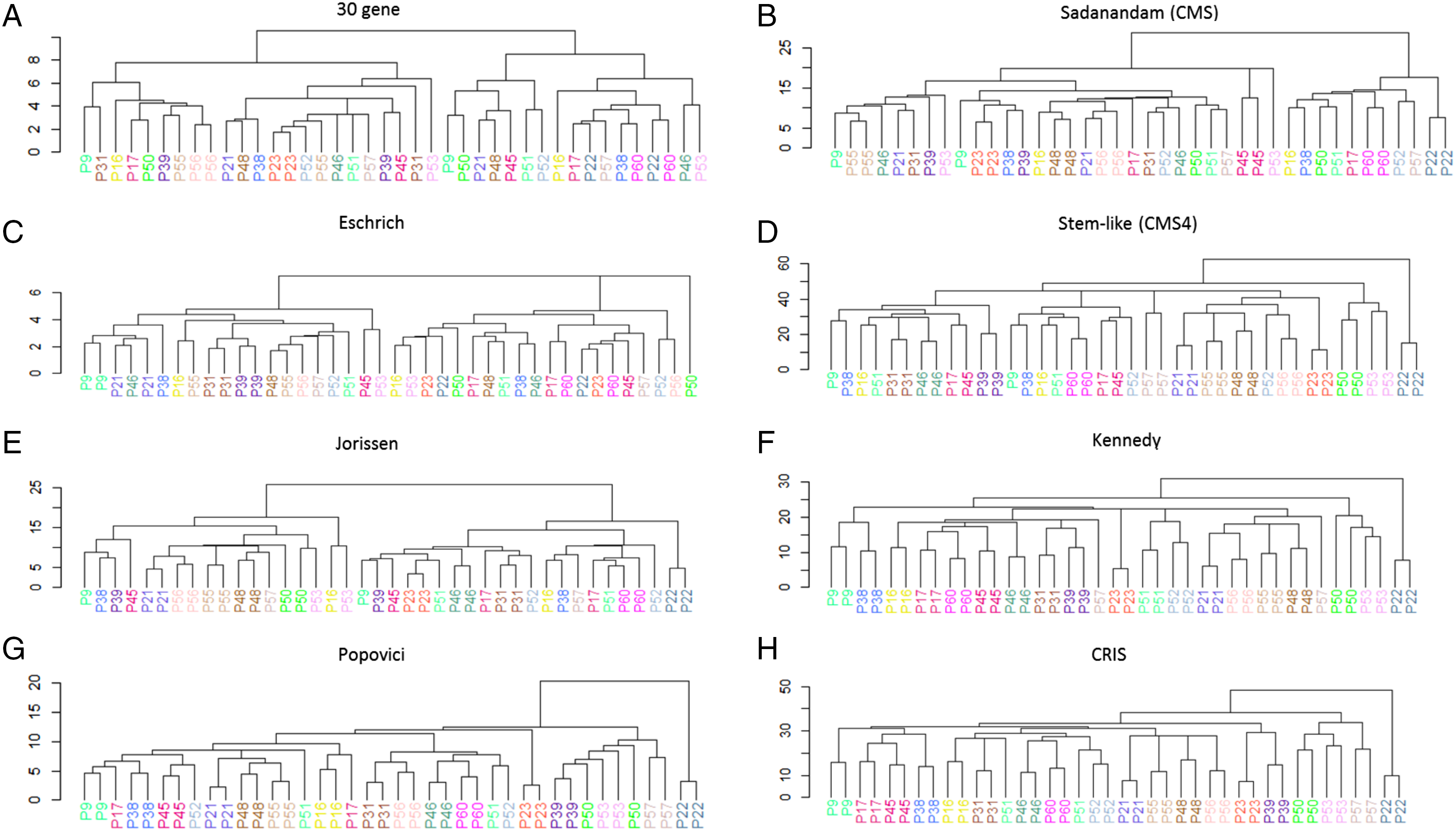

Supplement: Supplementary file 4 — Figure S3. Assessment of divisive clustering capabilities in matched CRC CT and IF regions using eight previously published CRC gene expression signatures. (A) 30 gene; (B) Sadanandam 5; (C) Eschrich 17; (D) stem‐like (CMS4); (E) Jorissen 16; (F) Kennedy 18; (G) Popovici 19; and (H) CRIS 9. [file PATH-245-19-s003.tif]

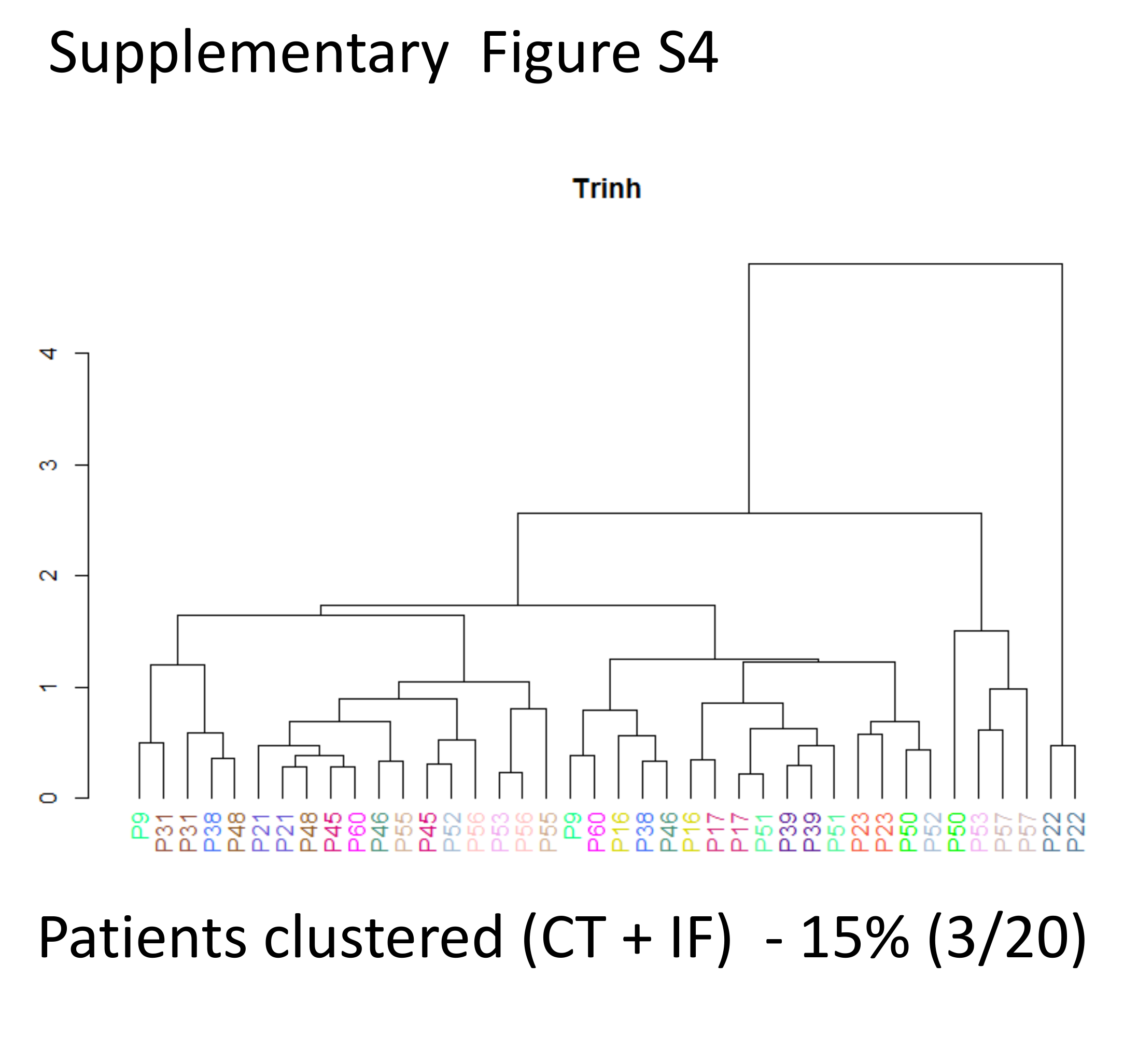

Supplement: Supplementary file 5 — Figure S4. Assessment of the clustering capabilities of the refined CMS classifier published by Trinh et al. 20. Divisive analysis clustering in matched CRC CT and IF regions using the Trinh gene expression signature. [file PATH-245-19-s004.tif]
